# A pilot, prospective evaluation of a novel alternative for maintenance therapy of breast cancer-associated lymphedema [ISRCTN76522412]

**DOI:** 10.1186/1471-2407-6-84

**Published:** 2006-03-29

**Authors:** Olivia Wilburn, Paul Wilburn, Stanley G Rockson

**Affiliations:** 1 Stanford Center for Lymphatic and Venous Disorders, Stanford University School of Medicine, Falk Cardiovascular Research Center, Stanford, California 94305, USA

## Abstract

**Background:**

Prospective investigations of complete decongestive lymphatic physiotherapy (CDPT), including manual lymphatic drainage (MLD), have validated the efficacy of these interventions for the initial reduction of edema and long-term maintenance of limb volume in lymphedema. However, CDPT demands substantial time and effort from patients to maintain these benefits; the treatments are not always well-accepted, and patients may suffer from a deterioration in quality-of-life or a time-dependent loss of initial treatment benefits. A new device designed for home use by the patient, the Flexitouch™, has been developed to mechanically simulate MLD. We have undertaken a prospective, randomized, crossover study of the efficacy of the Flexitouch™, when compared to massage, in the self-administered maintenance therapy of lymphedema.

**Methods:**

A prospective, randomized, crossover study of maintenance therapy was performed in 10 patients with unilateral breast cancer-associated lymphedema of the arm. Each observation phase included self-administered treatment with the Flexitouch™ or massage, 1 hour daily for 14 days, respectively, followed by crossover to the alternate treatment phase. Each treatment phase was preceded by a 1 week treatment washout, with use of garment only. The sequence of treatment was randomly assigned. The potential impact of treatment modality on quality of life was assessed with serial administration of the SF-36.

**Results:**

Statistical analysis disclosed that the order of treatment had no outcome influence, permitting 10 comparisons within each treatment group. Post-treatment arm volume reduced significantly after the Flexitouch™, but not after self-administered massage. The patients' mean weight decreased significantly with Flexitouch™ use, but not with massage. The Flexitouch™ device was apparently well-tolerated and accepted by patients. Serial SF-36 administration showed no deterioration in physical or psychosocial scores compared to baseline measurements; there were no statistical differences in scores when the two treatment modalities were compared.

**Conclusion:**

This short-term prospective evaluation of the Flexitouch™ suggests that the device may provide better maintenance edema control than self-adiminstered massage in breast cancer-associated lymphedema. The apparent ease of use and reliability of response to the device suggest that further broad-scale testing is warranted.

## Background

Acquired disruption of the lymphatic circulation can lead to either regional or generalized accumulation of interstitial fluid, known as lymphedema [1]. Among the predicted 200,000 patients who develop breast cancer annually [2], conservative estimates dictate that 15–20% will ultimately develop secondary lymphedema of the upper extremity [3,4]. The cumulative incidence of lymphedema appears to increase with each year after surgery [5]. The advent of arm lymphedema after breast cancer therapies engenders severe psychological and physical morbidity [6-8]. Lymphedema affects social relations, and undermines body image and self-esteem [9].

Complete decongestive physiotherapy (CDPT), a combined treatment program that incorporates manual lymph drainage, multi-layer short-stretch bandaging, exercise and skin care, has been recommended by a consensus panel of experts [10] and is considered to be the standard approach to lymphedema management [11-13]. Various of the elements of CDPT, while costly and labor-intensive [14], have demonstrable efficacy, with reported initial reductions of the measured excess limb volume that approximate 50% [15,16]. Nevertheless, despite the documented efficacy of initial, intensive CDPT in the hands of the trained therapist, it has been observed that the most important factor to influence the long-term impact of lymphedema interventions is the patient's subjective response to the management variables [17,18]. The time-consuming, labor-intensive, and repetitive nature of the treatment strategies pose difficulty and set the stage for poor, or incomplete, patient compliance [19]. Thus, the chronic response to physiotherapeutic lymphedema interventions, once patient self-management supervenes, is often characterized by a time-dependent loss, to varying degrees, of the beneficial effects of the short-term intensive interventions undertaken by medical personnel [15,16]. This treatment recidivism likely reflects the combined influences of patient inefficiency with the demanding techniques of manual lymphatic drainage and bandaging, and diminished compliance with self-management strategies.

Optimal management may have the capacity to slow the inexorable tendency of chronic lymphedema to progress, and may forestall or eliminate many of the recognized complications of the disorder, including recurrent soft-tissue infection and chronic fibrosis [10]. To this end, we have prospectively evaluated the potential benefit of a new approach to the maintenance phase of self-management in breast cancer-associated lymphedema.

The Flexitouch™ is a lightweight, portable device for home use that has been bio-engineered to closely simulate the effects of optimally performed manual lymphatic drainage. The device has been commercially available in the United States since June of 2002, when the Flexitouch™ system received 510 K clearance from the Food and Drug Administration. The current investigation was designed to evaluate the relative efficacy of the Flexitouch™, when compared to standard measures, in the maintenance self-management of breast cancer-associated lymphedema.

## Methods

### Design of the trial

**Figure 1:**
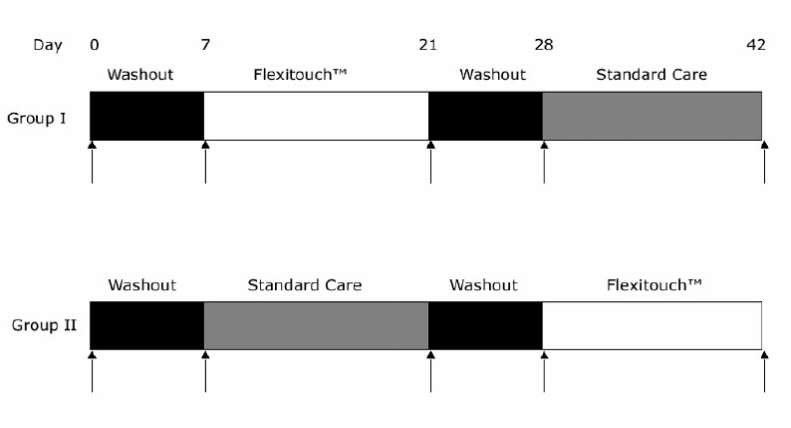
Schematic of the study protocol. There was a randomized assignment to Group I or II. During the washout periods, treatment was limited to the daily use of a compressive garment, with manual lymphatic drainage or other intervention. The vertical arrows indicate the times at which volumetry was performed by circumferential measurements of the limbs. The SF-36 was administered before and after each of the 14 day treatment phases.

A prospective evaluation of the Flexitouch™ in the maintenance management of breast cancer-associated lymphedema was undertaken in a prospective, randomized, crossover study. The investigation was undertaken from December 2003 through June 2004 and was approved by the Institutional Review Board of Stanford University. The study design is schematized in Figure 1.

### Patients

Patients with lymphedema of the upper extremity after surgical and/or radiotherapeutic interventions for breast carcinoma were eligible for enrollment. Recruitment was undertaken from the population of patients who presented to the Stanford Center for Lymphatic and Venous Disorders. Written informed consent was obtained from all participants.

### Inclusion and exclusion criteria

To be eligible for enrollment, a subject was required to have evidence of unilateral, breast cancer-associated lymphedema, with an increase of at least 10% in the measured volume of the affected arm when compared with the contralateral, normal limb. All subjects were required to have completed an initial treatment phase of limb volume reduction through intensive decongestive physiotherapy administered by a trained physiotherapist. A minimum of 30 days must have elapsed following the completion of initial treatment, which was required to include instruction in self-administered, maintenance simple manual lymphatic massage and the subsequent use of a properly fitted compression garment.

Exclusion criteria included the presence of any of the following: bilateral lymphedema of the upper extremity; active cancer; active infection; clinical evidence of venous obstruction or active thrombophlebitis; pulmonary edema; congestive heart failure; a history of pulmonary embolism; or the presence of any other relative contraindication to the use of the lymphedema treatment modalities employed in this investigation.

### Treatment allocation

Once enrolled in the study, subjects were allocated randomly, in equal numbers, to begin treatment with either the experimental approach (daily use of the Flexitouch™) or with use of standard treatment measures (compression garment plus daily self-administered massage). Cross-over to the alternate arm of therapy occurred in each enrolled patient after the designated observations and interval treatment washout had been completed. All study personnel responsible for patient observations and for data analysis were blinded to the subjects' treatment status, and patients were specifically instructed to refrain from discussing the particulars of their interval treatment status with the study personnel.

### Treatment methods

**Figure 2:**
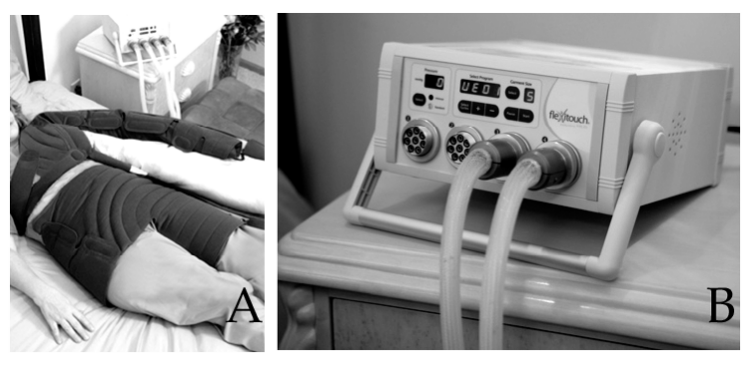
The figure depicts the unilateral Flexitouch™ garment as it is applied to the patient (A). The controller unit and connecting hoses are seen in panel B.

Patients were instructed in the standard, self-administered use of the Flexitouch™. The device provides 1–3 seconds of mild pressure that moves continuously through the device during an individual treatment. In theory, the device design should, by analogy to the purported mechanisms of manual lymphatic drainage, encourage excess interstitial fluid to follow existing pathways of lymph efflux from the trunk to regain access to the central circulation,. The device features a two-phase preparation and drainage program. The Flexitouch™ is comprised of an electronic controller unit, utilized in concert with specialized garments worn on the trunk and the affected upper extremity (Figure 2).

The patented garments include separate trunk and limb components that, when combined, consist of up to 32 separate chambers. The Flexitouch garment utilizes a proprietary composite of laminated materials that deliver variable stretch during inflation. This stretch is designed to simulate a therapist's hand movements during manual lymphatic drainage. The device delivers brief applications (1–3 seconds) of mild pressure in a continuously moving, rhythmic work-and release-action that is repetitively applied in each section. No two chambers are ever fully inflated at the same time.

The inflatable chambers of the garment, constructed of distensible fabric, connect to the controller unit by tubing harnesses. In the initial, lymph preparation phase, a modest gradient of pressure is applied to the trunk to prepare for, and direct, the augmented flux of lymph; thereafter, lymph drainage of the affected extremity is accomplished by concerted cycles of pressure applied sequentially to the limb and the trunk.

For the conventional treatment phase of the trial, self-administered massage and use of elastic compression garments was undertaken as patients had been previously instructed during the initiation phase of CDPT [16], prior to enrollment in this trial. Self-administered massage is advocated as a standard technique in the maintenance management of chronic lymphedema, with a demonstrated capacity to sustain at least a major component of the benefits derived from the active therapeutic interventions undertaken with a trained physiotherapist [16]. The use of any additional management strategies was prohibited during active participation in the trial.

### Treatment regimens

The active treatment phases were comprised of maintenance therapy with the Flexitouch™ alone or with self-administered massage alone, respectively, as an adjunct to the daily use of the compression garment. Each treatment modality was utilized for one hour daily during 14 consecutive days of treatment. Each phase of active treatment was preceded by a 1 week treatment washout, with use of the garment alone (no self-administered massage). The sequence of treatment was randomly assigned; the initial modality of therapy was followed, after a second washout phase, by crossover to the alternate treatment modality.

### Measurements and assessments

All outcomes measure and assessments were accomplished by medical personnel trained and experienced in the objective assessment and treatment of lymphedema.

#### Limb volume measurements

Limb volume was calculated by a validated, indirect volume measurement that utilizes surface measurements and a simplified formula derived from the formula for a frustum (truncated cone) [20]. This approach has been demonstrated to have excellent intra- and inter-observer reliability, and to yield results that are statistically indistinguishable from the water displacement method, accepted by many, but not all, as the 'gold standard' [21].

Both arms were measured at each time point. Serial measurements were performed using gauged tape to ensure a uniform stretching force. Limb circumferences were measured at axial intervals of 4 cm, beginning with an initial measurement at the radial styloid. For each patient, absolute limb volume was expressed in milliliters (ml). In addition, the volume of the edematous arm was expressed as a ratio to the volume of the contralateral, normal arm (Volume_Lymphedema_/Volume_Normal _× 100%).

#### Quality of life assessment

The SF-36 was serially administered to each enrolled patient, before and after each phase of active treatment. The SF-36 is a validated, generic instrument to assess, in broad terms, the impact of disease and its management upon various categories of psychosocial function [22]. This instrument has previously, successfully been applied to study of breast cancer-associated lymphedema [23].

#### Data analysis

In this study, continuous variables were statistically compared using analysis of variance, paired and unpaired *t*-test analysis (α = 0.05) and multiple regression analysis. For the sample size of the cohorts in this study and the primary endpoint of volume response, the statistical analysis had a power of 0.95 for an alpha = 0.01.

## Results

### Patient characteristics

Eleven (11) patients were approached for potential inclusion in this study. Consecutively presenting breast cancer survivors with unilateral upper extremity lymphedema were informed of the study at the time of their presentation to the Stanford Center for Lymphatic and Venous Disorders. Ten of these patients, all female, were enrolled in this prospective investigation.

The patient ages ranged from 54 to 78 years (60 ± 7 [mean ± SD]. For these patients, the duration since initial cancer therapy ranged from 36–288 months (103 ± 87 months). Onset of unilateral arm swelling was noted at 34 ± 34 months prior to enrollment in the study (range, 1–99 months). Initial cancer staging data was available for 8 of the 10 subjects: 2 initially presented with Stage I disease, 2 with Stage II and 3 with Stage III. Seven of the 10 subjects had undergone adjunctive radiotherapy.

There was an identical incidence of right- and left-sided involvement (5 subjects each) in this cohort. The mean pre-treatment body weight was 75 ± 12 kg (range, 58–83 kg). Hypertension (both systolic and diastolic) was prevalent in among the enrolled patients: the mean systolic pressure was 139 ± 14 mm Hg (120–167 mm Hg) and the mean diastolic pressure was 86 ± 7 mm Hg (80–95 mm Hg).

### Clinical responses

**Figure 3:**
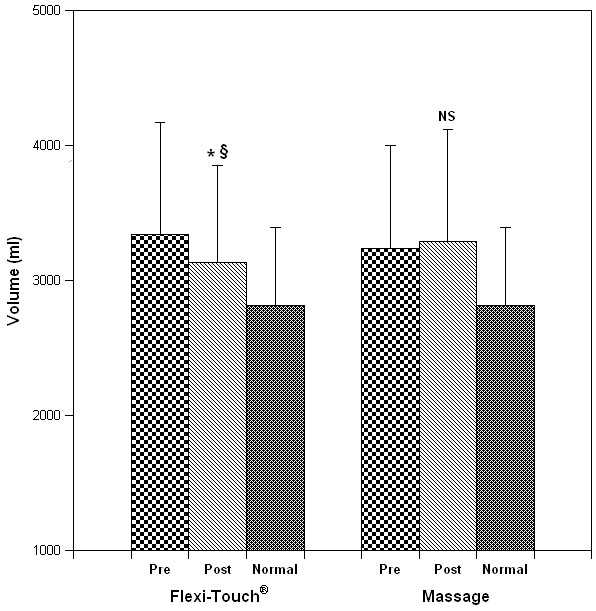
The effect of treatment modality on limb volume. Panels A and B depict the measured volume of the affected limb for each patient before and after Flexitouch™ and MLD therapy, respectively. Panel C illustrates the grouped data for each treatment phase (mean ± S.D.; * P = 0.002 when compared to pretreatment volume; § P = 0.007 when compared to the response to MLD). By multiple regression analysis, treatment status (Flexitouch™ *versus *massage) was predictive of volume change (P < 0.02). When controlled for the pre-treatment body weight, the results were unchanged; however, pretreatment arm volume was predictive of treatment response (P = 0.008).

**Figure 4:**
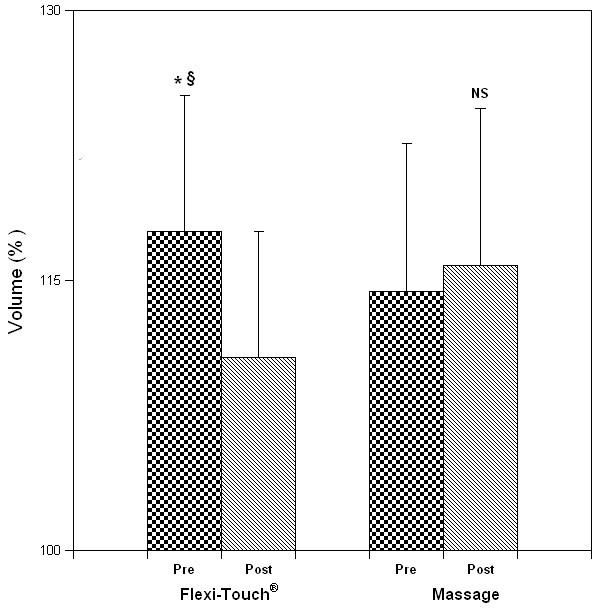
Excess volume of the affected arm, expressed as a percentage of the volume of the contralateral, normal arm. The effect of treatment on the % excess volume, when compared to the contra-lateral arm, was significant for Flexitouch™ but not for MLD (mean ± S.D.; *P = 0.0005 when compared to the pretreatment value; § P = 0.003 when compared to response to MLD).

**Figure 5:**
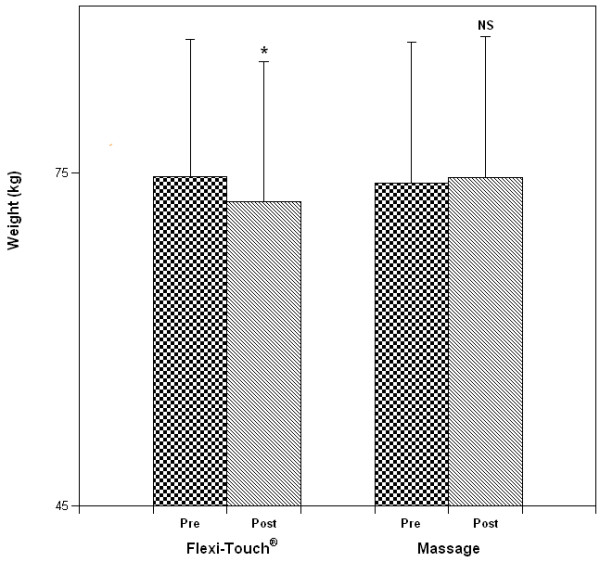
The effect of treatment strategy on body weight (mean ± S.D.; *P = 0.0002).

Five patients initiated the clinical evaluation with the Flexitouch™ treatment phase and 5 with self-administered massage, respectively. Statistical analysis disclosed no outcome influence of the order of treatment, permitting 10 comparisons within each treatment group. Arm volume reduced significantly from pre-treatment (Figure 3) after the Flexitouch™ (mean change, -208 ± 157 ml, P = 0.002) but not after self-administered massage (+52 ± 106 ml, NS); when the absolute volume differences following Flexitouch™ treatment were compared to those following self-administered massage, the reduction in volume attained with the device was statistically significant (P = 0.007). The effect of treatment on the % excess volume, when compared to the contralateral arm, was significant for Flexitouch™ (pretreatment, 15 ± 7% *vs*. post-treatment, 12 ± 6%, P = 0.0005) but not for self-administered massage (pre- and post-treatment, 14 ± 7%, NS) [Figure 4]. As a corollary effect of partial edema resolution, the patients' mean weight decreased significantly after Flexitouch™ (-2.3 ± 1.3 kg, P = 0.0002) but not after self-administered massage (+0.5 ± 0.9 kg, NS) [Figure 5]. For the sample size employed in this study and the primary endpoint of volume response, the statistical analysis had a power of 0.95 for an alpha = 0.01.

By multiple regression analysis, treatment status (Flexitouch™ *versus *massage) was predictive of volume change (P < 0.02). When controlled for the pre-treatment body weight, the results were unchanged; however, pretreatment arm volume was predictive of treatment response (P = 0.008). The change in limb volume with treatment was poorly correlated to the change in body weight (r = 0.3).

### Quality of life indicators

Serial administration of the SF-36 demonstrated neither deterioration nor improvement in physical or psychosocial scores when compared to baseline or to those referable to standard therapy with self-administered massage. The total scores for the SF-36 were 132 ± 34 (baseline), 133 ± 33 (Flexitouch™), and 131 ± 37 (massage), respectively. Analysis of variance disclosed no statistical differences.

In addition to the serial administration of the SF-36, subjects were directly queried about their experiences with the device. The Flexitouch™ treatment phase was, by patient report, universally well-tolerated and -accepted. No adverse experiences were reported at the time of follow-up, or thereafter.

## Discussion

With chronic obstructive derangement in the regional lymphatic circulation, an edematous state ensues that is characterized, over time, by chronic architectural alterations in the skin and supporting tissues [1,24]. Lymphedematous tissues are conceptualized to have a lower oxygen content, a greater distance between lymph vessels induced by the interstitial fluid accumulation, impaired lymphatic clearance, and depressed macrophage function, endowing these patients with an increased risk of soft tissue infection [25]. Once established, lymphedema has an inexorable tendency to progress. The concerted effects of increased interstitial fluid volume and chronic fibrotic changes expose these patients to substantial functional impairment, coupled with the psychosocial dysfunction that emanates from the chronic disease state and the associated impairment in body image and self-esteem [26-28].

Although the deleterious consequences of chronic lymphedema can be substantially reduced by effective physical management [10,15], the long-term impact of chronic decongestive physiotherapy upon lymphedema has not yet been optimized. While initial, intensive physiotherapeutic interventions can effectively minimize volume changes in the edematous limb, concerted self-management is required to maintain therapeutic benefits. Patients can effectively be taught to self-administer manual lymphatic drainage, but a gradual diminution in the magnitude of the initial therapeutic benefit is predictable [16]. It is likely that this phenomenon is related to some combined effect of patient inefficiency and a time-dependent reduction in patient compliance with self-management techniques. Adjunctive therapies, like intermittent pneumatic compression, have proven benefit [29], but may compound patient difficulty with compliance, since, of necessity, these adjunctive treatment measures lengthen the daily requirement for self-management.

In an attempt to improve the impact of maintenance self-management strategies on chronic lymphedema, we have undertaken the current study to investigate the relative efficacy of the Flexitouch™ when compared with a standard treatment approach (self-administered massage). The biodesign of the device and its impact on the disease state confer some distinct theoretical advantages. Because the Flexitouch™ is bioengineered to closely simulate the effects of correctly performed simple manual lymphatic massage, it should theoretically minimize the effect of improper or inadequate technique upon the treatment outcome. Furthermore, because the device emphasizes comfort and minimizes the active participation of the patient, it should, in principle, optimize compliance.

Indeed, our observations suggest that, even in short-term use, the device confers therapeutic benefit over that which can be attained through standard therapy. In addition to the demonstrable small, but significant, improvement in edema volume, the statistically significant reduction in body weight that accompanies the use of the Flexitouch™ serves as an independent confirmation of the added efficacy of this approach for the reduction of edema volume. While the magnitude of weight loss cannot be entirely explained by the quantitative reduction in limb edema volume, the concurrent changes seem to validate the beneficial impact of the Flexitouch™. The phenomenon of weight loss certainly bears additional investigation in larger numbers of patients. The current study was not designed to specifically address this response and, therefore, additional study with control of relevant variables during the treatment phase, such as fluid intake, diet, exercise, and medications, will be required to validate the observed phenomenon and to address the potential mechanisms of the therapeutic benefit.

The current, pilot study is limited chiefly by the small sample size and short duration of the therapeutic intervention. The positive results suggest that further investigation will be useful. When observed in larger cohorts of patients, with treatment phases sustained over longer intervals, it can be anticipated that even more substantial volume benefits might be demonstrable. In addition, quality-life-indicators should theoretically have the capacity to detect any favorable impact of streamlined therapy upon perceptions of the disease state. Finally, it will be desirable to attempt to demonstrate the theoretical favorable impact of this intervention upon tissue architecture and other chronic structural consequences of lymphedema. It is well-recognized that lymphedema, with chronicity, occasions the secondary proliferation of fibroblasts, keratinocytes, and adipocytes, the accumulation of collagen, and the destruction of elastin fibers within the skin [30]. It has been hypothesized that effective physiotherapy of lymphedema can diminish or eliminate these otherwise inexorable consequences. It is relevant that, in a recent study of the effect of therapeutic intervention upon lymphatic filariasis, the institution of even simple measures to reduce edema possessed the capacity to ameliorate the histopathological manifestations of chronic inflammation and fibrosis in punch biopsies of the skin [31]. While such direct histological confirmation may not be as readily derived from a breast cancer-associated lymphedema population, future, more extensive studies of the therapeutic potential of the Flexitouch™ might realistically incorporate other, more indirect attempts to document an ameliorative effect upon tissue architecture. Furthermore, evaluation of patients during more protracted use of the device, if it indeed enhances treatment outcome while minimizing the incursion of self-management upon the activities of daily living, would be predicted to disclose an improvement in perceptions of psychosocial and physical well-being. The aggregate benefits to be derived from this device will then require consideration in relationship to the retail cost of this device as it is currently marketed (unilateral garment, US $10,800; bilateral garment, US $12,400).

## Conclusion

Short-term prospective evaluation of the Flexitouch™ device suggests that it may provide better maintenance control of edema volume than simple manual lymphatic drainage in patients with breast cancer-associated lymphedema. The ease of use and reliability of response to the device suggest that further broad-scale testing is warranted. Economic implications of the treatment choices will ultimately also be required to assess its relative merits within the scope of maintenance lymphedema care.

## Abbreviations

CDPT: Complete decongestive lymphatic therapy

MLD: Manual lymphatic drainage

SD: Standard deviation

## Competing interests

Tactile Systems, Inc., the manufacturer of the Flexitouch™, generously made available, on loan, the Flexitouch™ devices utilized in the current study. The authors have no financial ties to the company. Tactile Systems had no role in the design, execution, analysis, or communication of this investigation.

## Authors' contributions

OW participated in the study design, completed data collection, and participated in the drafting of the manuscript; PW participated in data collection and analysis; SGR conceived of the study, participated in its design and coordination, assisted in statistical analysis and drafted the manuscript. All authors read and approved the final manuscript.

## Pre-publication history

The pre-publication history for this paper can be accessed here:
